# Endoplasmic reticulum stress in perivascular adipose tissue promotes destabilization of atherosclerotic plaque by regulating GM-CSF paracrine

**DOI:** 10.1186/s12967-018-1481-z

**Published:** 2018-04-18

**Authors:** Ru Ying, Sheng-Wei Li, Jia-Yuan Chen, Hai-Feng Zhang, Ying Yang, Zhen-Jie Gu, Yang-Xin Chen, Jing-Feng Wang

**Affiliations:** 1 Department of Cardiology, Sun Yat-sen Memorial Hospital of Sun Yat-sen University, No.107, Yanjiang West Road, Yuexiu District, Guangzhou, 510120 China; 2 Department of Cardiology, The First Affiliated Hospital of NanChang University, Nanchang, 330006 China; 3 Department of Respiratory Medicine, The 94th Hospital of Chinese People’s Liberation Army, Nanchang, 330026 China

**Keywords:** Perivascular adipose tissue, Endoplasmic reticulum stress, Atherosclerosis, Vulnerable plaque, Granulocyte Macrophage Colony Stimulating Factor

## Abstract

**Background:**

Perivascular adipose tissue (PVAT) accelerates plaque progression and increases cardiovascular risk. We tested the hypothesis that PVAT contributed to plaque vulnerability and investigated whether endoplasmic reticulum stress (ER stress) in PVAT played an important role in vulnerable plaque.

**Methods:**

We transplanted thoracic aortic PVAT or subcutaneous adipose tissue as a control, from donor mice to carotid arteries of recipient apolipoprotein E deficient (apoE^−/−^) mice after removing carotid artery collar placed for 6 weeks. Two weeks after transplantation, ER stress inhibitor 4-phenyl butyric acid (4-PBA) was locally administrated to the transplanted PVAT and then animals were euthanized after 4 weeks. Immunohistochemistry was performed to quantify plaque composition and neovascularization. Mouse angiogenesis antibody array kit was used to test the angiogenic factors produced by transplanted adipose tissue. In vitro tube formation assay, scratch wound migration assay and mouse aortic ring assay were used to assess the angiogenic capacity of supernatant of transplanted PVAT.

**Results:**

Ultrastructural detection by transmission electron microscopy showed transplanted PVAT was a mixed population of white and brown adipocytes with abundant mitochondria. Transplanted PVAT increased the intraplaque macrophage infiltration, lipid core, intimal and vasa vasorum neovascularization and MMP2/9 expression in plaque while decreased smooth muscle cells and collagen in atherosclerotic plaque, which were restored by local 4-PBA-treatment. Antibody array analysis showed that 4-PBA reduced several angiogenic factors [Granulocyte Macrophage Colony Stimulating Factor (GM-CSF), MCP-1, IL-6] secreted by PVAT. Besides, conditioned medium from 4-PBA treated-PVAT inhibited tube formation and migration capacity of endothelial cells and ex vivo mouse aortic ring angiogenesis compared to conditioned medium from transplanted PVAT. mRNA expression and protein levels of GM-CSF were markedly elevated in adipocytes under ER stress which would be suppressed by 4-PBA. In addition, ER stress enhanced NF-κB binding to the promoter of the mouse GM-CSF gene in adipocytes confirmed by Chromatin immunoprecipitation analyses.

**Conclusions:**

Our findings demonstrate that ER stress in PVAT destabilizes atherosclerotic plaque, in part through increasing GM-CSF paracrine via transcription factor NF-κB.

**Electronic supplementary material:**

The online version of this article (10.1186/s12967-018-1481-z) contains supplementary material, which is available to authorized users.

## Background

Acute coronary syndrome (ACS) is the leading cause of cardiovascular morbidity and mortality. Most major adverse events occur independent of the plaque size and the degree of luminal stenosis and most ACS arises from vulnerable plaques which are more prone to rapid plaque progression [1]. The vulnerable lesion has several structural and functional hallmarks different from stable plaque including large necrotic core, inflammation, thin fibrous cap, depletion of smooth muscle cells and collagen, and increased formation of neo microvessels [2]. However, the precise mechanisms underlying plaque destabilization are still unclear.

Accumulating evidences have shown a strong association between obesity and cardiovascular disease [3]. Obesity is associated with the inflammation in perivascular adipose tissue (PVAT) which not only serves a structural support for most arteries but also secretes series of molecules to actively modulate vascular function [4]. In 1991, it was the first time to report that PVAT had an anticontractile function [5]. In 2002, PVAT was found to release a vasoactive factor [6]. Since then, the list of factors released from PVAT has expanded and now includes adipokines (e.g., leptin and adiponectin), angiogenesis factors and inflammatory factors (e.g., MCP-1, IL-6). From twentieth century on, scientists revealed that chemokines production from PVAT played a role in the pathogenesis of atherosclerosis [7]. These molecules might contribute to the alterations of the function and structure of vascular wall, including chronic inflammation, alterations of vascular tone, smooth muscle cell dysfunction, neo-angiogenesis and hence to the development of atherosclerosis. Epidemiological studies show that PVAT correlates with plaque burden in human [8–10]. Experimental animal studies also demonstrate that PVAT accelerated atherosclerosis in mice [11, 12]. Additionally, clinical observations suggest that coronary perivascular adipose tissue is related to the presence of lipid core, macrophage infiltration and severity of atherosclerotic plaque which are the characteristics of high risk plaque [13, 14]. These findings urged us to hypothesize that PVAT contributes to plaque vulnerability through paracrine effects on the vasculature from ‘outside to inside’.

There are compelling evidences that endoplasmic reticulum stress (ER stress) plays fundamental roles in atherogenesis and atherosclerotic progression [15, 16]. Endoplasmic reticulum (ER) is the cellular organelle in which protein folding, calcium homeostasis, and lipid biosynthesis occurs [15]. Perturbations in ER homeostasis cause ER stress. High fat feeding and obesity [17, 18] and several other high-risk factors of atherosclerosis such as hypertension [19, 20], cigarette smoking [21], high glucose [22] and hyperhomocysteinemia [23], can lead to increased ER stress in adipose tissue. ER stress induces inflammatory phenotypic alteration of adipose tissue which plays an important role in atherogenesis [17]. Thus, we speculated that unresolved ER stress might induce the dysfunction of PVAT by leading to aberrant chemokines secretion and contributed to atherosclerotic plaque progression.

In this study, we found that transplanted PVAT promoted plaque vulnerability in the setting of high-fat diet (HFD) which could be ameliorated by 4-PBA at least in part dependent on decreased GM-CSF released locally by transplanted PVAT. These findings demonstrate a direct relationship between ER stress in PVAT and plaque destabilization and implicate GM-CSF secretion by PVAT as a mediator of this pathological process.

## Methods

### Animal model

Eight weeks old male apoE^−/−^ mice (Guangdong medical laboratory animal center) were used. To investigate the impact of PVAT on atherosclerotic stability, we executed carotid collar placement as described [24, 25] and these mice were maintained on a HFD (Guangdong medical laboratory animal center, 0.15% cholesterol and 21% fat). The carotid collar was removed after 6 weeks, followed by adipose tissue transplantation surgery. 2 weeks after transplantation, ER stress inhibitor 4-PBA (5 μg per mouse) was locally administrated using pluronic gel to the transplanted PVAT and then animals were euthanized 4 weeks later. The left common carotid arteries and the transplanted adipose tissue were collected for histological and molecular biological analysis.

All procedures were approved by the Institutional Animal Care and Use committee of Sun Yat-sen University at Guangzhou.

### Carotid collar placement

A silastic tube (0.3 mm inner diameter, 0.5 mm outer diameter, and 2.5 mm long) was placed around the left common carotid near its bifurcation. Briefly, mice were anesthetized and the left common carotid arteries were dissected from the surrounding connective tissue. Collars were placed carefully around the left carotid arteries and then tied with three circumferential silk ties at their axial edges. Then, the entry wound was closed.

### Adipose transplantation to carotid artery

Thirty mg of PVAT was collected from the thoracic aorta of donor C57BL/6J mice (Guangdong medical laboratory animal center) fed a HFD for 4 weeks. The collected PVAT, or inguinal subcutaneous adipose tissue (SQAT) from the same group of donor mice as a control, was implanted adjacent to left common carotid artery of apoE^−/−^ mice. Sham-operated apoE^−/−^ mice underwent the same surgery without a fat transplant. Skin was sutured with 6-0 nylon filament.

### Angiogenesis-related protein analysis

To analyze the expression profiles of angiogenesis-related proteins, we used the mouse angiogenesis antibody array (RayBiotech, USA), according to the manufacturer’s instructions. They can detect 24 antibodies directed to proteins involved in angiogenesis. Briefly, 10 mg tissue of PVAT and SQAT were incubated in 1 ml serum-free DMEM medium. After 24 h the supernatants from tissue cultures were centrifuged at 14,000 rounds/min for 3 min. The centrifuged supernatants were stored at − 80 °C until further processing.

Supernatants from tissue cultures were mixed with 70 μl of biotin-conjugated detection antibodies for 2 h at room temperature with gentle shaking. Following a washing step, streptavidin-fluor was added to each sub-array. Make sure the glass slides were absolutely dry before scanning or storage. Data were captured by GenePix 4000B Microarray Scanner (Molecular Devices, USA) and analyzed by analysis tool software for RayBio antibody array (RayBiotech).

### Cell culture

Mouse preadipocytes 3T3-L1 cells (Shanghai institute for biological sciences, Shanghai, China) were cultured in growth medium (DMEM supplemented with 10% FCS). Two days postconfluence, the cells were induced to differentiate with standard cocktail consisting of medium (DMEM supplemented with 10% FBS) with 1 μmol/l dexamethasone, 10 μg/ml insulin and 0.5 mmol/l isobutyl-methylxanthine (Sigma). After 4 days in differentiation medium, the cells were treated with medium containing 10 μg/ml insulin for 4 days and then maintained in DMEM supplemented with 10% FBS alone. Then, cells were considered mature adipocytes. ER stress was induced by tunicamycin (TM) and suppressed by 4-PBA.

Human umbilical vein endothelial cells (HUVECs) (Shanghai institute for biological sciences) were maintained in endothelial cell growth medium-2 (EGM-2; Lonza) supplemented with 10% FBS (Biological Industries, Israel), at 37 °C in humidified incubator (5% CO_2_). HUVECs were cultured to 2–6 passages for experiment.

### Mouse aortic ring assay

Three-dimensional ex vivo mouse aortic ring angiogenesis assay allows analysis of cellular proliferation, migration, tube formation, microvessel branching, perivascular recruitment and remodeling, providing a more complete picture of angiogenic processes compared with traditional in vitro tube formation assays. We performed the aortic ring assay as previously described [26], with minor modifications. Briefly, the rings from the aortas of 4 weeks old C57BL/6 mice were inserted between two layers of matrigel basement membrane matrix (BD Biosciences) and cultured in EGM-2 in the presence of DMEM or supernatants from PVAT cultures for 7 days. Endothelial sprouts were stained with antibody to CD31 (abcam) by immunofluorescence and quantitative analysis was performed with software Image-Pro Plus 6.0. Three separate aortic sections were quantified for each aorta, and the results were averaged for each animal.

### In vitro tube formation assay

96-well plates were coated with 50 μl Matrigel (Millipore) and incubated at 37 °C for 60 min to allow the Matrigel to solidify. HUVECs were plated at a density of 5 × 10^4^ cells/well with supernatants from PVAT cultures or vehicle and incubated at 37 °C for 8 h. The cells were then photographed using a Nikon digital camera. Five randomly selected view fields were photographed in each well. The average of five fields was taken as the value for each sample. Tube formation was quantified by measuring the length of capillary structures using the software Image-Pro Plus 6.0 [27].

### Cell migration assay

The scratch wound migration assay was performed as described before [28]. Briefly, confluent HUVEC sheets were starved for 6 h before starting the experiments. Confluent cell monolayer was then scraped with a 200 μl pipette tip to generate scratch wounds and rinsed twice with PBS. Cells were photographed immediately and 24 h after the scratch with a Nikon digital camera (Nikon ECLIPSE Ti). The wound area was then measured to determine cell migration.

### Chromatin immunoprecipitation (ChIP)

ChIP analysis was performed using Chromatin IP kit (Pierce). Anti-p65(pNF-κB) antibody (CST 8242S) was used for immunoprecipitation and then pNF-κB association with GM-CSF promoters was estimated using SYBR-Green RT-PCR. For positive control polymerase II antibody was used (Cell Signaling Technology). Corresponding normal IgG was used as negative control. ChIP was performed overnight at 4 °C with rotation. Input DNA was purified along with ChIP probes. Association with pNF-κB and GM-CSF promoters was estimated using SYBR-Green RT-PCR. Sequence of primers used for ChIP is given in Additional file 1: Table S1.

### Ready-To-Glow™ NF-κB Secreted Luciferase Reporter System

We used the Ready-To-Glow™ NF-κB Secreted Luciferase Reporter System (Clotech, Catalog No.631743), according to the manufacturer’s instructions. Mature 3T3-L1 cells were transiently transfected with pMetLuc2-control vector or pNFκB- MetLuc2-reporter vector. 24 h after transfection, the media was removed and replaced by media with vehicel or TM (1 μg/ml) or pretreatment of 4-PBA (5 mM) for half hour to activate the NF-κB signal transduction pathway. After 8, 16 and 24 h, 160 μl media samples were removed and analyzed for Metridia luciferase activity in a luminometer (Molecular devices, SpectraMax M5/M5e). The fold induction was calculated for different time points following substrate addition.

### Statistical analysis

The statistical significance of differences between groups was determined by one-way ANOVA for multiple comparisons, or Student’s *t* test when comparisons were made between two groups. Values are expressed as mean ± SEM, *p *< 0.05 are considered significant.

## Results

### Effects of adipose tissue transplantation

**Fig. 1:**
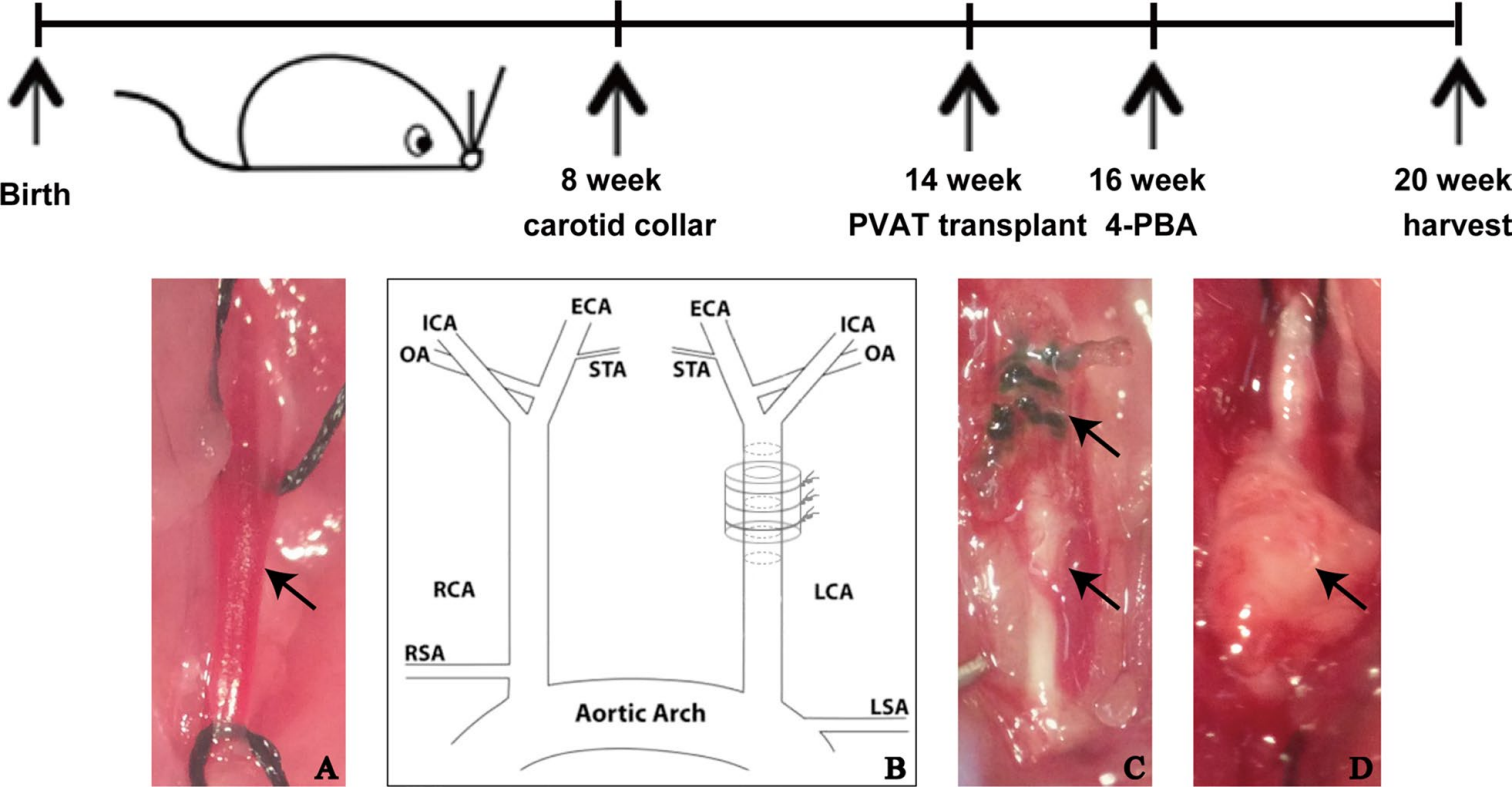
Schematic showing experimental procedure with surgical timeline of adipose tissue transplantation. **A** Left common carotid artery of sham operation group. **B** Schematic diagram of carotid collar placement. **C** Effect of carotid collar placement. **D** Effect of adipose tissue transplantation

To determine whether adipose tissue could contribute to characteristics of atherosclerotic plaque, ApoE^−/−^ mice underwent PVAT, SQAT transplant or sham operation to the left common carotid artery a site that is devoid of perivascular adipose tissue and typically does not develop spontaneous atherosclerosis. The surgical images showed that transplanted adipose tissue was healthy appearing and was incorporated into the carotid adventitia (Fig. 1).

**Fig. 2:**
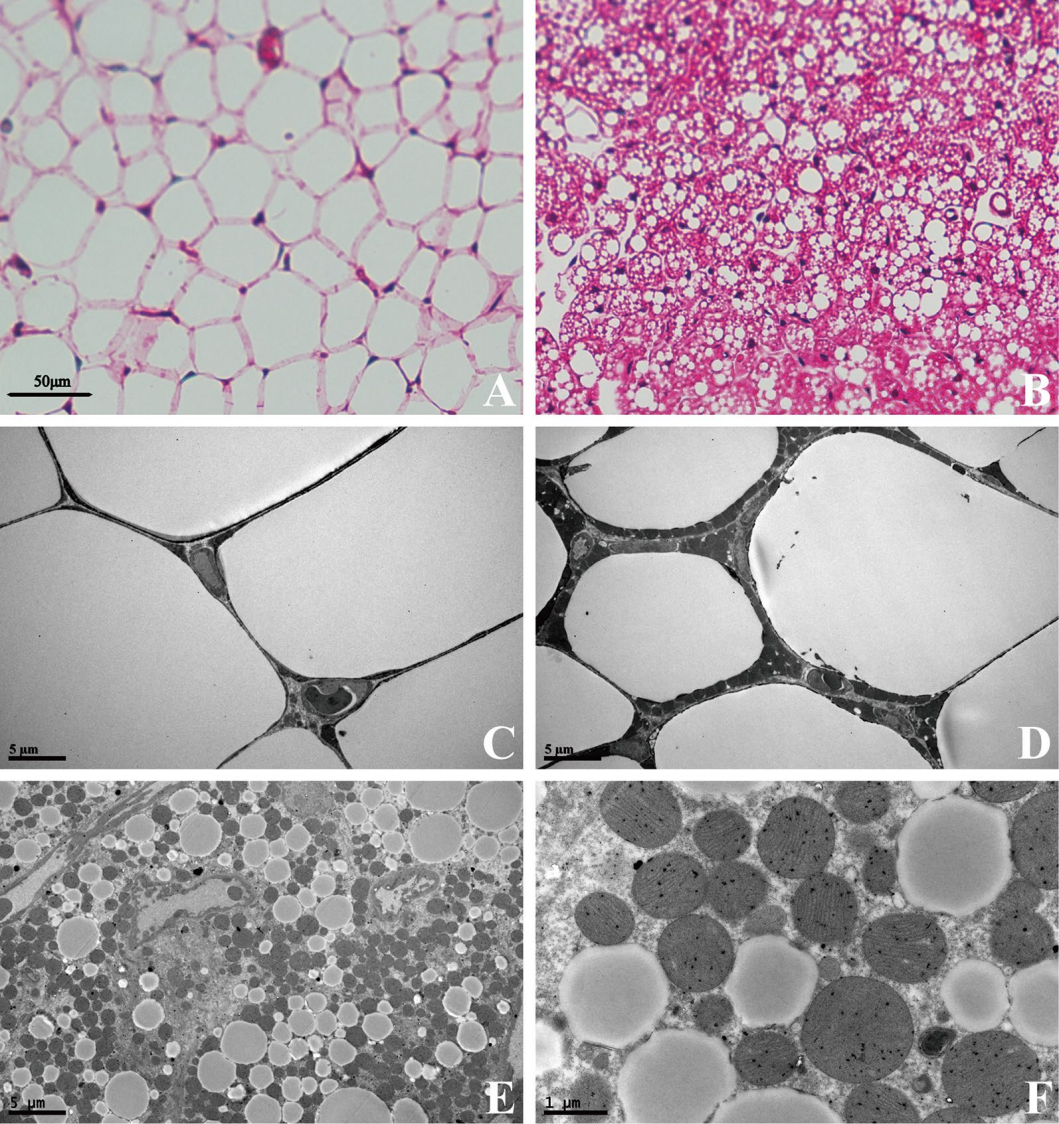
Histology of adipose tissue. **A** HE staining of SQAT. **B** HE staining of PVAT. **C** Ultrastructural detection of SQAT by transmission electron microscopy at ×2400 magnification. **D** PVAT on transmission electron microscopy at ×2400 magnification. **E** Another section of PVAT on transmission electron microscopy at ×2400 magnification. **F** PVAT in **E** on transmission electron microscopy at ×13,500 magnification

HE staining demonstrated that SQAT was white adipocytes while PVAT was similar to brown adipocytes (Fig. 2A, B). However, ultrastructural detection by transmission electron microscopy showed PVAT was a mixed population of white and brown adipocytes (Fig. 2D–F) and the adipocytes had abundant mitochondria with one big or several small lipid droplets in PVAT while SQAT was made up of a single lipid vacuole with rare mitochondria (Fig. 2C).

To exclude the systemic confounding factors, we compared mouse weight, systemic lipid levels and serum adipokines of PVAT-transplanted animals with mice in other groups and found that there were no significant difference between them (Additional file 1: Table S2). In addition, analysis of gene expression showed that mRNA expression of leptin and adiponectin in transplanted PVAT and SQAT were similar to endogenous fat from the corresponding depots of the same mice, which suggested that adipose phenotype was not affected by the transplantation experiment (Additional file 1: Figure S1).

### Effects of transplanted adipose tissue on destabilization of atherosclerotic plaque

**Fig. 3:**
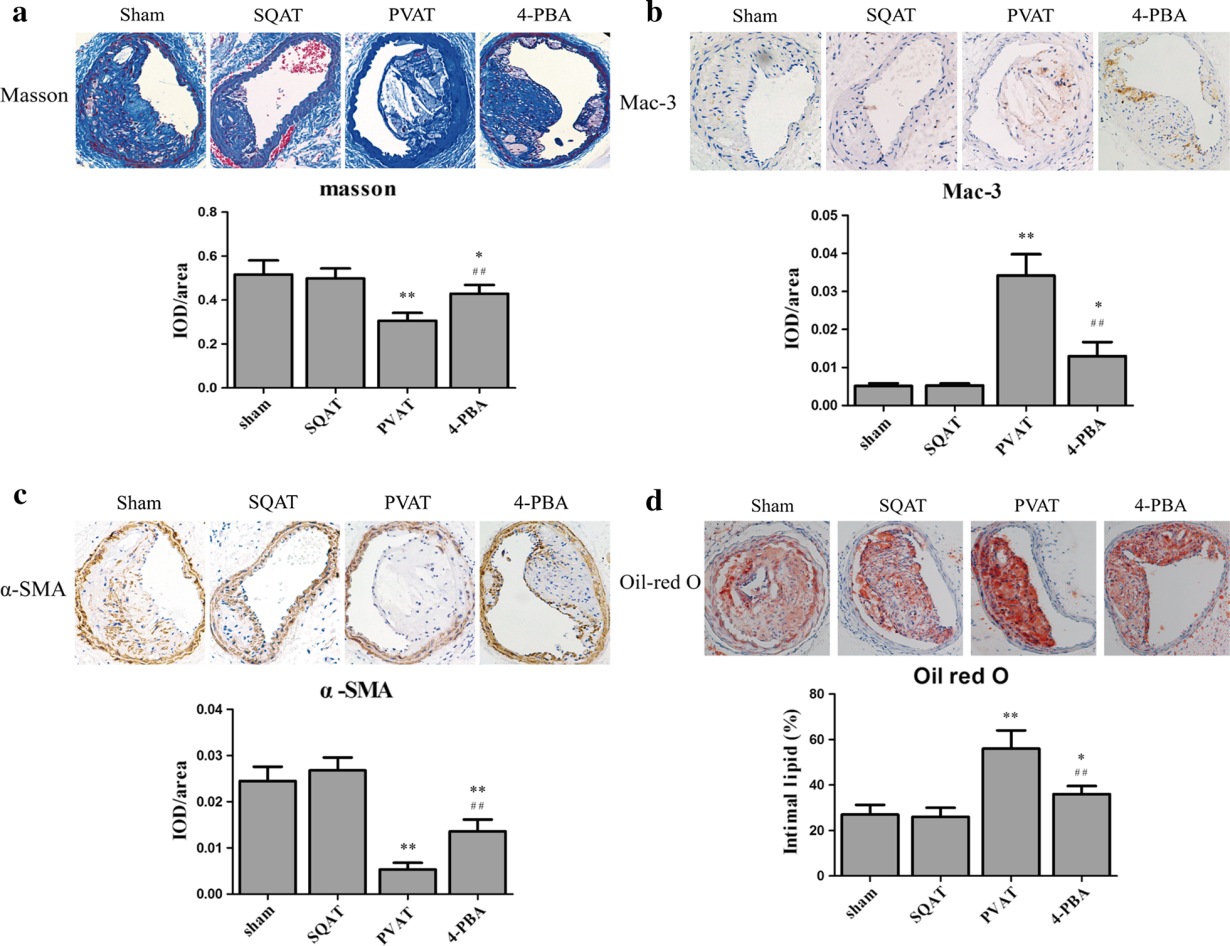
Effects of adipose tissue transplantation on plaque composition. **a** Masson Trichrome staining was used to detect collagen and fibrin in plaque. **b**, **c** Immunostaining for mac-3/α-SMA was performed for intraplaque macrophages/SMCs. **d** Oil red O staining for lipid core in plaque. n = 6. *p < 0.05, **p < 0.01 compared with sham group, ^#^*p *< 0.05, ^##^*p *< 0.01 compared with PVAT group

To characterize the effects of PVAT on plaque composition, collagen, SMCs, macrophages and cholesterol core of carotid artery lesion were detected. Masson Trichrome is a three-color staining protocol used in histology, which stains collagen and bone into blue or green, keratin and muscle fibers into red, cell nuclei into dark brown to black and cytoplasm into light red or pink. Oil red O is a fat-soluble dye used for staining of lipids into red color. PVAT transplantation markedly increased intraplaque macrophages number, enlarged lipid core and upregulated MMP2/9 expression compared to SQAT transplantation while reduced collagen and SMCs in plaque (Fig. 3 and Additional file 1: Figure S2). Especially, the fibrous cap mainly composed of collagen and SMCs in PVAT group was thinner than that in sham and SQAT group (Fig. 3a). Moreover, SQAT transplantation failed to substantially change the plaque composition.

### Effects of 4-PBA treatment on plaque composition

To investigate whether endoplasmic reticulum stress, a characteristic of obese adipose tissue, plays a role in the effects of PVAT determined above, ER stress inhibitor 4-PBA was locally administrated to the transplanted PVAT. After ER stress in PVAT was inhibited, intraplaque collagen and SMCs number increased compared to PVAT group without inhibiting ER stress while intraplaque macrophage infiltration, lipid core and MMP-2 expression decreased (Fig. 3 and Additional file 1: Figure S2). However, 4-PBA failed to decrease the expression of MMP-9 in plaque.

### Effects of transplanted adipose tissue on intimal and vasa vasorum neovascularization

**Fig. 4:**
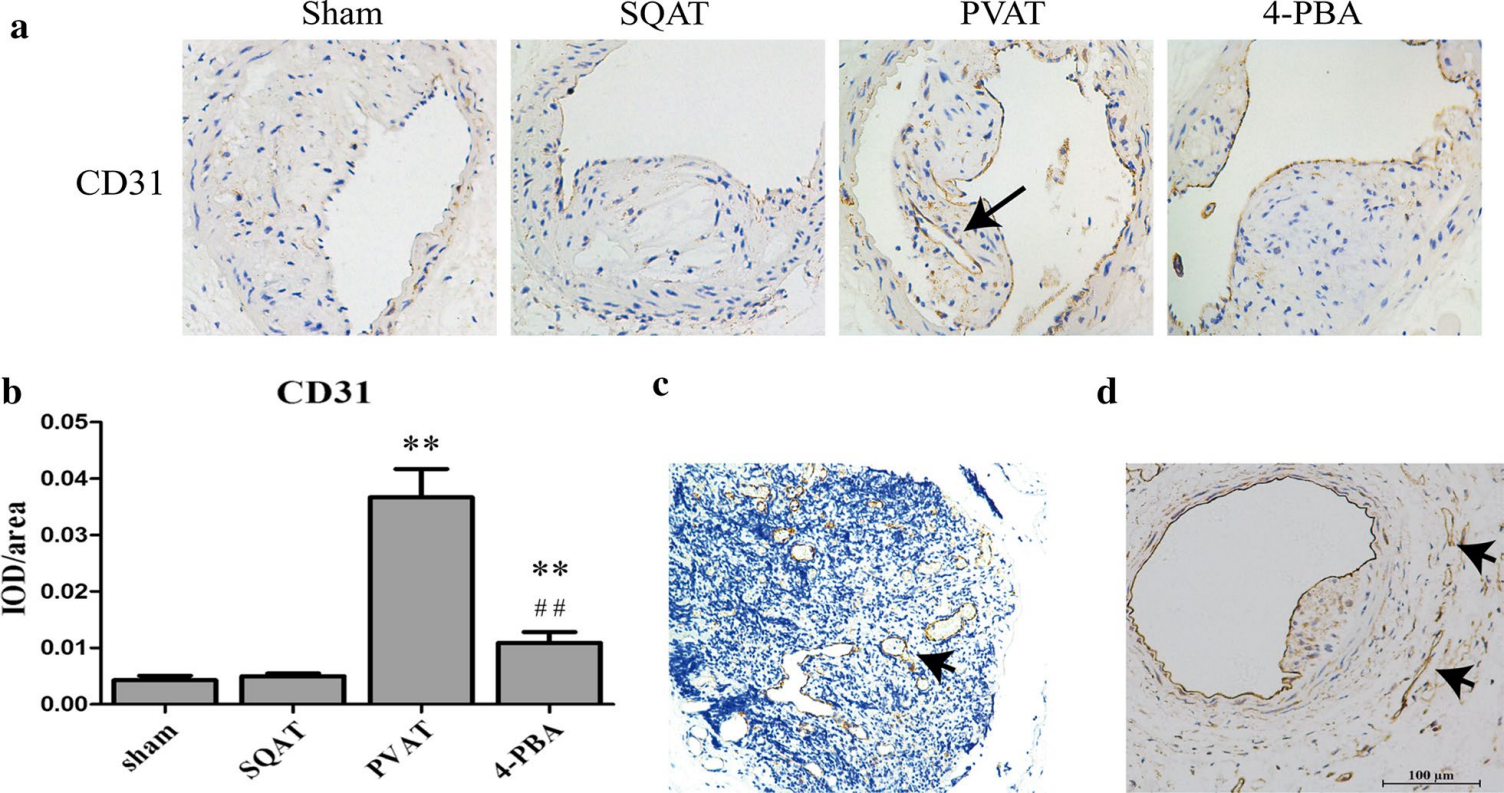
Effects of transplanted adipose tissue on intimal and vasa vasorum neovascularization. **a** Immunostaining for CD31 was performed to evaluate intraplaque angiogenesis. **b** Statistical analysis for **a** (n = 6). **p < 0.01 compared with sham group, ^##^*p *< 0.01 compared with PVAT group. **c** Immunostaining for CD31 in another mouse in PVAT group, suggesting markedly neovascularization and thrombus organization. **d** Vasa vasorum neovascularization of the mice in PVAT group

Intraplaque angiogenesis functions to sustain the growth of subintima and media from the artery lumen, and vasa vasorum can facilitate macrophage and erythrocyte entry into the plaque and cause inflammation and intraplaque hemorrhage [29]. Therefore, intimal and vasa vasorum neovascularization would destabilize plaque. The mice in sham group and SQAT group had no obvious intraplaque microvessel development, while transplanted PVAT promoted intraplaque angiogenesis (Fig. 4a, c). When ER stress was inhibited in transplanted PVAT, microvessels were significantly reduced in plaque (Fig. 4d). Likewise, vasa vasorum (VV) were not present in mice of sham group and SQAT group, but transplanted PVAT increased VV neovascularization (Fig. 4d) which would be attenuated by 4-PBA.

### Conditioned medium from PVAT promoted tube formation and migration capacity of endothelial cells and ex vivo mouse aortic ring angiogenesis

**Fig. 5:**
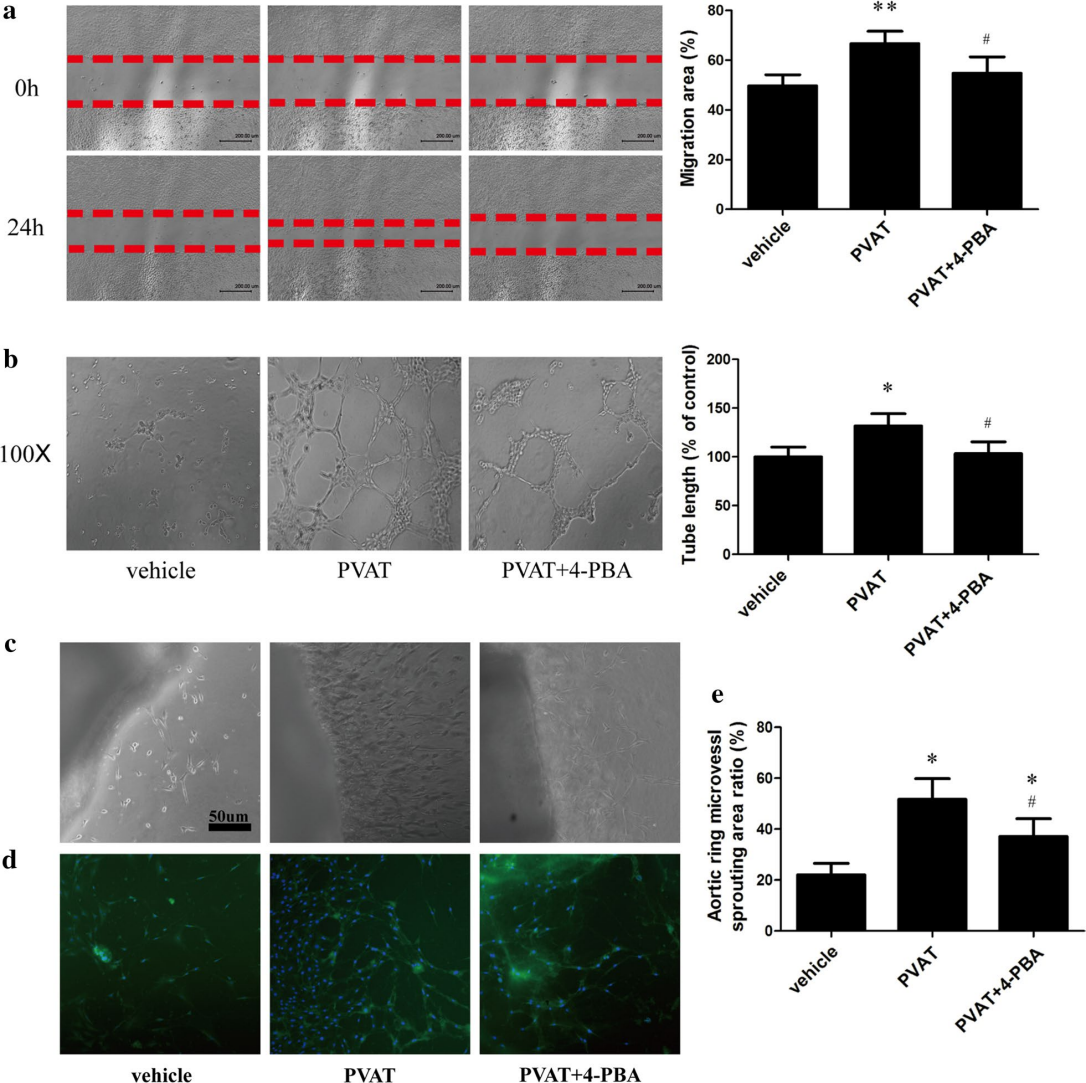
Conditioned medium from PVAT promoted angiogenesis in vitro and ex vivo. **a** Scratch wound migration assay. **b** *In vitro* tube formation assay. **c** Ex vivo mouse aortic ring angiogenesis. **d** Immunostaining for CD31 of mouse aorta in **c**. **e** Statistical analysis for **c** (n = 6). *p < 0.05 compared with vehicle group, **p < 0.01 compared with vehicle group, ^#^*p *< 0.05 compared with PVAT group

Although PVAT transplantation increased in vivo intraplaque and vasa vasorum neovascularization, whether it could up-regulate angiogenesis in vitro and ex vivo need to be further investigated. We used in vitro tube formation assay and scratch wound migration assay to assess tube formation and migration capacity of endothelial cells. The conditioned medium from transplanted adipose tissue were used to administrate endothelial cells. The migration of ECs treated with the supernatant of transplanted PVAT was faster than control group and the tube formation capacity of the former was also stronger than the latter. Moreover, supernatant of 4-PBA treated-PVAT would restored tube formation and migration function of ECs (Fig. 5a, b).

We next tested ex vivo angiogenesis via mouse aortic ring assay. The supernatant of transplanted PVAT markedly promoted the ex vivo mouse aortic ring angiogenesis which was confirmed by immunostaining of CD31 (Fig. 5c, d). When ER stress in PVAT was inhibited by 4-PBA, the angiogenesis effect would become weaker.

Thus, from the in vitro, ex vivo and in vivo evidences, we concluded that PVAT could promote angiogenesis, which could be attenuated by ER stress inhibitor.

### Mouse angiogenesis antibody array for angiogenic factors produced by transplanted adipose tissue

**Fig. 6:**
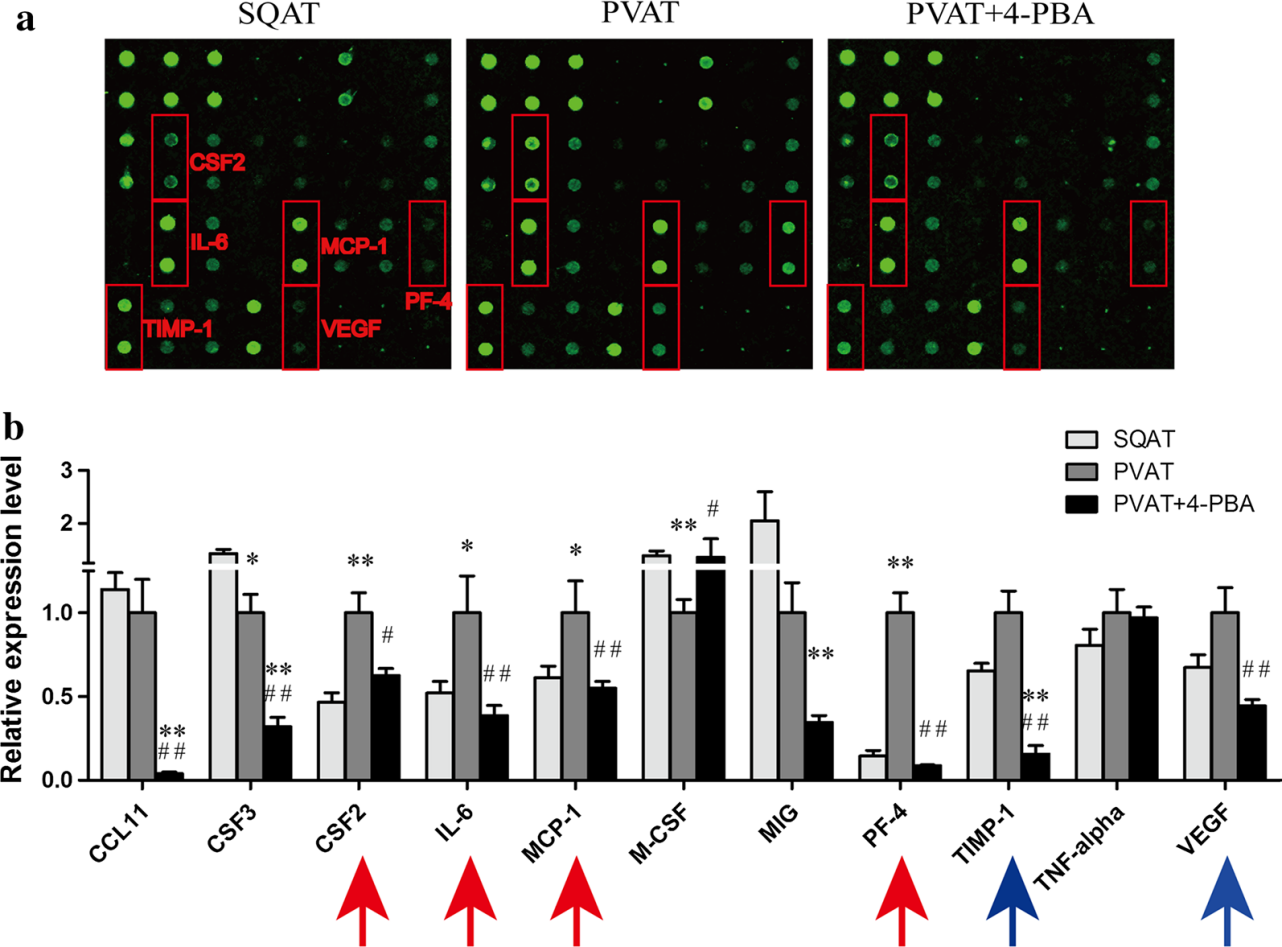
Mouse angiogenesis antibody array for supernatant of transplanted adipose tissue. **a** Mouse angiogenesis antibody array detected 24 antibodies. **b** Statistical analysis for **a** (n = 3)

In spite of angiogenic effect of PVAT, it is still unkown about the related angiogenic factors playing an important role in the angiogenic process. Therefore, we determined to screen out these factors by mouse angiogenesis antibody array which could detect 24 antibodies directed to proteins involved in angiogenesis. The results suggested that PVAT increased several pro-angiogenic factor levels (MCP-1, IL-6, GM-CSF) and also up-regulated the expression of anti-angiogenic factor (PF-4) (Fig. 6).

### ER stress upregulated GM-CSF expression of adipocytes by a transcriptional mechanism

**Fig. 7:**
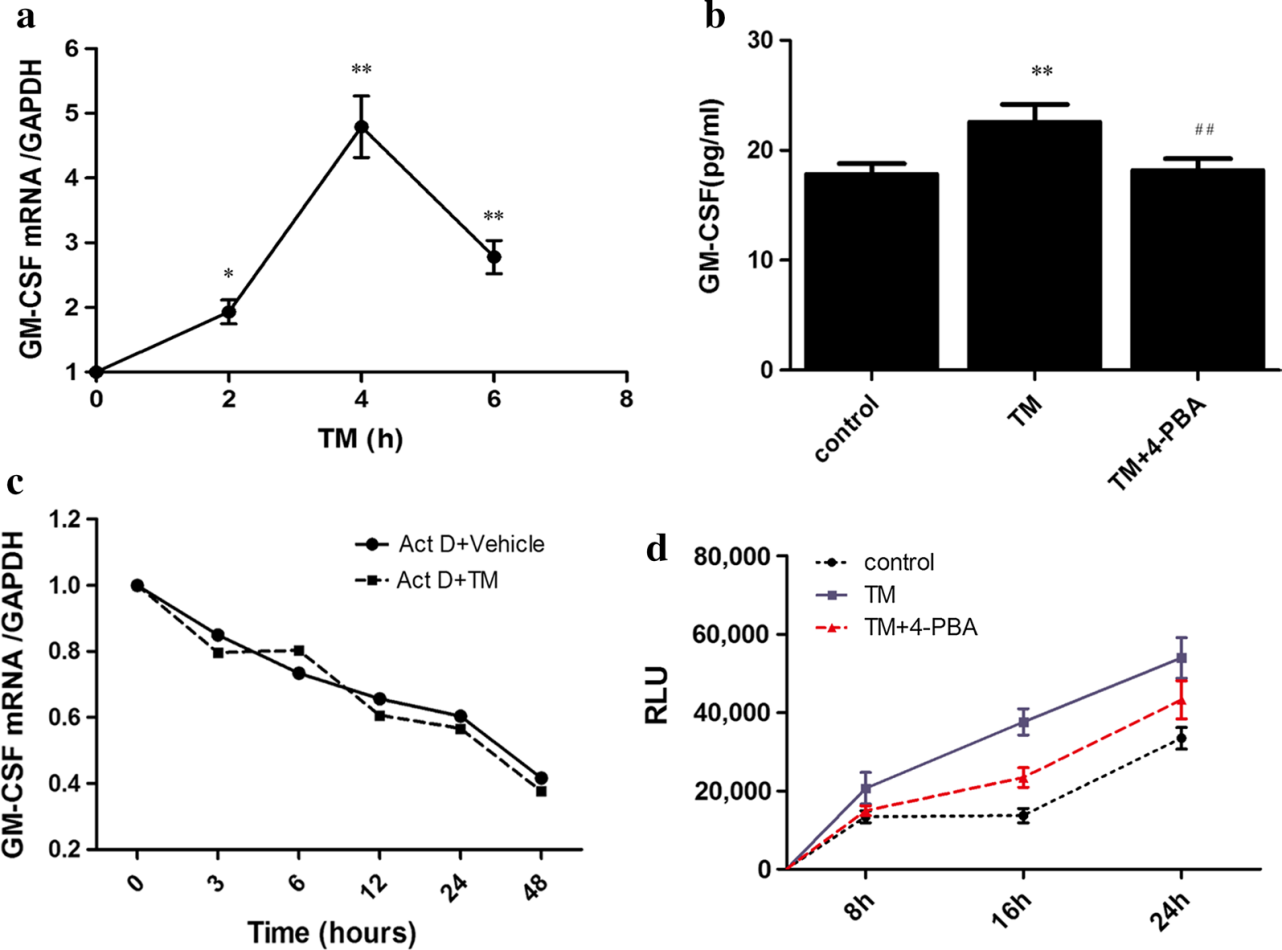
ER stress upregulated GM-CSF expression by a transcriptional mechanism. **a** GM-CSF mRNA levels of adipocytes treated with TM (1 μg/ml) in different time. **b** Elisa results of supernatant of adipocytes treated with TM in the presence or absence of 4-PBA. **c** RT-PCR for GM-CSF mRNA assessment. Adipocytes were administrated with 5 μg/ml Actinomycin D in the presence or absence of 1 μg/ml TM. **d** Ready-To-Glow™ NF-κB Secreted Luciferase Reporter System to assess NF-κB activity

The results of angiogenesis antibody array revealed that 4-PBA reduced GM-CSF expression produced by PVAT. Then, we established the models of ER stress in adipocytes. We treated adipocytes with ER stress inducer tunicamycin (TM) (1 μg/ml) or vehicle (DMSO) in the presence or absence of 5 mM 4-PBA. QRT-PCR results showed that TM induced GM-CSF gene expression in 3T3-L1 adipocytes and peaked at the 4th hour (Fig. 7a). Elisa results suggested the supernatant of adipocytes treated by TM had higher GM-CSF level than control, and 4-PBA attenuated GM-CSF expression (Fig. 7b).

Next, we investigated the mechanism of ER stress regulating GM-CSF expression was transcriptional or posttranscriptional. For this purpose, adipocytes were administrated with the transcriptional inhibitor Actinomycin D (5 μg/ml) in the presence or absence of 1 μg/ml TM. RT-PCR results revealed that TM treatment failed to prolong the half-life of GM-CSF mRNA, which suggested that ER stress upregulated GM-CSF by a transcriptional mechanism (Fig. 7c).

One candidate transcription factor that could explain the effects of ER stress on GM-CSF is NF-κB. Therefore, we used Ready-To-Glow™ NF-κB Secreted Luciferase Reporter System to assess NF-κB activity. NF-κB was more active in TM treated-adipocytes than in control group, but some of this activity was lost when the cells were pretreated with 4-PBA, which demonstrated that NF-κB might contribute to ER stress induced GM-CSF expression.

### NF-κB contributed to increasing GM-CSF mRNA expression by ER stress

**Fig. 8:**
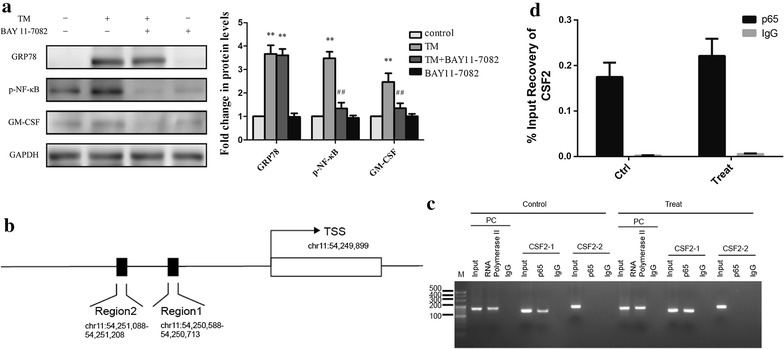
NF-κB contributed to the increase in GM-CSF mRNA by ER stress. **a** Western blot for pNF-κB and GM-CSF. Adipocytes were pretreatment of BAY11-7082 for half hour and then incubated with TM for 24 h (n = 3). **b** The two predicted site for NF-κB binding to GM-CSF (CSF2). **c** ChIP measurement. CSF2-1 and CSF2-2 were two predicted binding sites. **d** Results of NF-κB binding to site 1 (*p *< 0.05) (n = 3)

To verify whether NF-κB contribute to GM-CSF expression induced by ER stress, we conducted studies with NF-κB inhibitor BAY11-7082 (10 μM) and confirmed it by western blot. Results showed that BAY11-7082 inhibited NF-κB phosphorylation, and attenuated GM-CSF expression by TM (Fig. 8a).

To further confirm whether NF-κB could bind to the promoter of GM-CSF, we measured ChIP at two predicted binding sites. ChIP results showed that NF-κB could bind to the predicted region 1, but failed to binding site 2 (Fig. 8b, c). TM treatment increased occupancy of NF-κB at the binding site 1 in GM-CSF promoter (Fig. 8d).

## Discussion

A consensus is emerging that PVAT is related to plaque vulnerability, but direct evidence is lacking. Here, we have established a model in which PVAT was transplanted to carotid artery with atherosclerosis to test the effects of PVAT transplantation on plaque vulnerability. In addition, we locally administrated 4-PBA to the transplanted PVAT to investigate the role of ER stress in paracrine effects of PVAT. Our data suggest that ER stress in PVAT could increase plaque vulnerability, partly by increasing GM-CSF paracrine via transcription factor NF-κB.

Adipose tissue not only stores energy but also secrets numerous growth factors, cytokines, and hormones. PVAT has recently been recognized as a novel factor in vascular biology implicated in the pathophysiology of cardiovascular disease. Emerging researches suggest that PVAT may contribute to the pathogenesis of vascular disease by producing large numbers of biologically active molecules [6]. Our study may explain its biological activity from the perspective of the microstructures. Even though PVAT was a mixed population of white and brown adipocytes, ultrastructural detection by transmission electron microscopy showed that there were abundant big mitochondria no matter with small lipid droplets or one big lipid droplet in PVAT while SQAT was made up of a single lipid vacuole with rare mitochondria (Fig. 2). Therefore, the rich mitochondria in perivascular adipocytes might be the structure basis of its biological activity.

Vulnerable plaque is an important culprit mechanism for the development of acute coronary syndrome (ACS), which is influenced by several factors such as vasa vasorum and intraplaque neovascularization, large necrotic core, inflammation and intraplaque protease activity [2]. However, the underlying mechanism of plaque vulnerability remains unclear and need to be further elucidated. Clinical observations suggest PVAT increases severity of atherosclerotic plaque and correlates with coronary plaque burden [13]. We demonstrated PVAT promoted plaque vulnerability in PVAT-transplanted carotid arteries (Figs. 3, 4 and Additional file 1: Figure S2). To examine the impact of PVAT, endogenous PVAT in femoral artery was removed and replaced with transplanted fat in some studies [30, 31], or exogenous PVAT was transplanted to the carotid artery without PVAT in other studies [11, 12]. Fat transplantation was used in our study to reduce injuring artery. Carotid collar placement was used to study plaque vulnerability and produced atherosclerosis plaque in fixed location and facilitate administration to artery.

Several researches have also revealed the pathogenic role of PVAT in atherosclerosis [32–34]. David Manka et al. [11] transplanted PVAT to the mouse carotid artery and found that PVAT could enhance the neointimal response to vascular injury. On the contrary, some studies suggest that PVAT is vasculoprotective. For instance, removal of endogenous PVAT enhanced the neointimal response to wire injury in the femoral artery [30]. Additionally, research in the SMPG knockout mouse suggest an atheroprotective effect of PVAT at a cool temperature [35]. However, the results in current study are not strictly comparable with the previous researches. Some of these researches transplanted visceral adipose tissue rather than real PVAT to the carotid artery. Some studies removed endogenous PVAT, which might make the artery injured. Therefore, these conclusions might be dependent on the experimental model. However, the model of fat transplantation used in this research might be more proportionate to study the impact of PVAT.

Several high-risk factors of atherosclerosis can cause PVAT dysfunction and increase ER stress in adipose tissue [7]. In current research, we locally treated the transplanted PVAT with ER stress inhibitor 4-PBA and found that reduction ER stress in PVAT would attenuate the effect of PVAT on plaque destabilization and angiogenesis (Fig. 3, 4 and Additional file 1: Figure S2). Thus, we speculated that ER stress might contribute to the dysfunction of adipose tissue and lead to aberrant adipokine secretion.

Intimal angiogenesis, as a source of intraplaque hemorrhage, is associated closely with plaque vulnerability. From the in vitro, ex vivo and in vivo evidences, we concluded that PVAT could increase angiogenesis which would be attenuated by ER stress inhibitor. So then, we screened out the angiogenesis factors produced by transplanted adipose tissue with mouse angiogenesis antibody array kit. The results showed that several factors might play a role in the paracrine effect of PVAT, mainly including MCP-1, IL-6 and GM-CSF (Fig. 6). We focused on GM-CSF, because there were several reports on the role of MCP-1 and IL-6 secretion in PVAT.

GM-CSF is a hematopoietic growth factor, stimulating hematopoietic stem cells to produce granulocytes and monocytes. It is also a proinflammatory and pro-angiogenic factor. Additionally, GM-CSF could recruit and activate M1 macrophages and thus contributes to adipose tissue inflammation in response to a HFD [36]. The current data indicate that 4-PBA impaired production of GM-CSF in PVAT accompanied by improved plaque stabilization. Interestingly, another study also found that GM-CSF promoted plaque vulnerability due to promotion of macrophage apoptosis and plaque necrosis [37]. In addition, several studies suggest GM-CSF is correlated with plaque burden [38–40]. Therefore, GM-CSF might play an important role in the impact of PVAT on plaque vulnerability. However, this conclusion was based on the previous study and direct evidence is needed for better understanding the link between GM-CSF and plaque vulnerability.

Next, we used the transcriptional inhibitor Actinomycin D to demonstrate that ER stress upregulated GM-CSF expression in adipocytes by a transcriptional mechanism. Previous researches found that ER stress activated transcriptional factor nuclear factor-κb (NF-κB) [41, 42] and other studies suggested NF-κB was a transcription factor of GM-CSF [43, 44]. NF-κB is a transcription factor that controls the expression of genes involved in immune responses, inflammation, and cell cycle, which can be activated by a variety of stimuli, including cytokines, T and B cell mitogens, viral proteins, and stress inducers [45]. Therefore, we hypothesized that ER stress might increase NF-κB binding to the promoter of GM-CSF gene in adipocytes. Our present results showed that NF-κB could bind to the promoter of GM-CSF in the absence of stimulation, and the combination would be enhanced by ER stress inducer. Thus, NF-κB was a transcription factor of GM-CSF in adipocytes. However, 3T3-L1 cell line was used in this part of research, which might make the significance of the results limited.

## Conclusion

This is the first study to show that ER stress in PVAT promoted atherosclerotic plaque vulnerability by increasing GM-CSF paracrine via NF-κB. Moreover, the results provide an insight into the novel therapy of 4-PBA against ER stress-induced GM-CSF secretion in PVAT. Thus, therapeutic interventions that reduce ER stress in PVAT may be promising strategies to treat acute coronary disease. Epicardial adipose tissue injection of 4-PBA in patients undergoing coronary artery bypass grafting or via thoracoscope might have therapeutic implication. In addition, nano-targeted delivery of 4-PBA to PVAT might be a novel method for the treatment of vulnerable plaque in the future.

## Additional file

**Additional file 1.** PVAT promotes destabilization of atherosclerotic plaque.

## Abbreviations

PVAT: perivascular adipose tissue
ER Stress: endoplasmic reticulum stress
SQAT: subcutaneous adipose tissue
apoE^−/−^: apolipoprotein E deficient
4-PBA: 4-Phenyl butyric acid
GM-CSF: Granulocyte Macrophage Colony Stimulating Factor
ChIP: Chromatin immunoprecipitation
ACS: acute coronary syndrome
ER: endoplasmic reticulum
HFD: high-fat diet
HUVECs: human umbilical vein endothelial cells
TM: tunicamycin
EGM-2: endothelial cell growth medium-2
