# Supplementary material for: Endoplasmic reticulum stress in perivascular adipose tissue promotes destabilization of atherosclerotic plaque by regulating GM-CSF paracrine
Source: J Transl Med. 2018 Apr 18;16:105. doi: 10.1186/s12967-018-1481-z (PMC5907173; doi:10.1186/s12967-018-1481-z)
Supplement: Supplementary file 1 — Additional file 1. PVAT promotes destabilization of atherosclerotic plaque. [file 12967_2018_1481_MOESM1_ESM.docx]

Endoplasmic reticulum stress in perivascular adipose tissue promotes destabilization of atherosclerotic plaque by regulating GM-CSF paracrine

**Supplemental Material**

**Materials and Methods**

**Serum lipids, adipokines and GM-CSF analysis**

Blood samples from mice were collected by cardiac puncture under anesthesia of chloral hydrate after 8-h fast. Total cholesterol (TC), triglycerides (TG), high density lipoprotein-cholesterol (HDL-C), low density lipoprotein-cholesterol (LDL-C), adiponectin and leptin were measured by commercially available murine ELISA kits (BlueGene Biotech). The supernatant GM-CSF levels of adipocytes were also investigated by ELISA kit (Neobioscience).

**Ultrastructural detection by transmission electron microscopy**

In brief, adipose tissue was fixed in 2% glutaraldehyde overnight at 4°C, followed by 1% glutaraldehyde and 1% osmic acid for 2h. Specimens were dehydrated with acetone and embeded and cut into sections of 70nm in thickness. Ultra-thin tissue was examined with a JEM-2100 (HR) electron microscope (JEOL Ltd., Tokyo, Japan).

**Immunohistochemistry**

Immunohistochemistry was performed to characterize lesion composition. Carotid artery cross sections were stained with antibodies to Mac-3 (BD Biosciences, San Jose, CA) and α-smooth muscle actin (α-SMA) (Abcam) to quantify macrophages and smooth muscle cells (SMCs), respectively. Masson Trichrome staining (Sigma-Aldrich, St Louis, MO) was used to quantify fibrin and connective tissue elements in the plaque. Immunostaining for MMP2, MMP9 (Abgent) and CD31 (Abcam) was also performed in paraffin-embedded. Oil red O staining was carried out using frozen sections.

**Western blot assay**

Cells or tissue were lysed with RIPA (Roche) in the presence of protease inhibitor mixture (Roche) and incubated for 20 min at room temperature. The cell lysate was centrifuged for 30 min at 14,000 g at 4 °C. Supernatant was stored at -80 °C until further use. The total protein concentration was determined using the BCA Protein Quantitation Kit (Biocolors, China). Equal amounts of protein extracts were separated by gel electrophoresis using a 10% SDS-PAGE gel and transferred to polyvinylidene fluoride (PVDF) membranes (Millipore). The membrane was blocked for 1 h at room temperature in 5% BSA and immunoblotted at 4 °C overnight with the anti-GRP78 antibody (Abcam), anti-GM-CSF antibody (Abcam), anti-pNFκB antibody (CST). Detection was performed using with an ECL advanced system (Syngene).

**Quantitative Real Time PCR**

mRNA levels of leptin, adiponectin and GM-CSF were determined by quantitative Real-Time PCR. In brief, total RNA was extracted with TRIzol reagent (Invitrogen). RNA was reverse transcribed to cDNA by using an PrimeScript^TM^ RT reagent kit (TaKaRa). Reverse transcription was performed at 37°C for 15 min and 85°C for 5 sec. The resulting cDNAs were PCR amplified using SYBR® Premix Ex TaqTM (TaKaRa). The PCR primer sequences were listed in Tab.S3.

**Tables**

**Tab.S1** Sequence of primers used for ChIP

| Name | F | R |
| --- | --- | --- |
| GAPDH promoter | CATGGGTGTGAACCATGAGA | GTCTTCTGGGTGGCAGTGAT |
| GM-CSF promoter-1 | GCCTGGGAGAACTTGCCAG | ACATAGTAACTAGCTGTAACACAATAACCAGG |
| GM-CSF promoter-2 | CCTGTTATCTGACCCTCGAAAGC | GTGCCCTCTTGATATGAGAGGG |

**Tab.S2** Body Weight, Serum Lipids and Serum adipokines (n=6)

|  | sham | SQAT | PVAT | PVAT+4-PBA |
| --- | --- | --- | --- | --- |
| Body weight, g | 30.5+3.3 | 31.0+3.2 | 31.1+2.8 | 30.6+3.3 |
| TC, mmol/l | 14.8+3.6 | 15.0+2.1 | 15.3+2.0 | 14.5+2.4 |
| TG, mmol/l | 4.54+0.49 | 4.73+0.61 | 4.69+0.35 | 4.64+0.38 |
| LDL-C, mmol/l | 9.64+1.97 | 10.71+1.63 | 9.80+2.76 | 11.28+1.59 |
| HDL-C, mmol/l | 3.33+0.38 | 3.81+0.76 | 3.41+0.58 | 3.63+0.33 |
| Leptin, ng/ml | 17.92+2.91 | 18.96+5.23 | 16.88+3.39 | 17.19+2.63 |
| Adiponectin,μg/ml | 12.01+1.57 | 11.02+2.67 | 10.09+1.64 | 12.16+1.93 |

TC (total cholesterol), LDL-C (low density lipoprotein cholesterol), HDL-C (high density lipoprotein cholesterol), TG (triglycerides).

**Tab.S3** RT-PCR primer sequences

| Name | Sense primer | Antisense primer |
| --- | --- | --- |
| GAPDH | GTGGCAAAGTGGAGATTGTT | CTCGCTCCTGGAAGATGG |
| GM-CSF | AGA TAT TCG AGC AGG GTC TAC | GGG ATA TCA GTC AGA AAG GTT |
| leptin | AGACAGTGAGCCCCAAGAAA | GGAACAAAACTCCCCACAGA |
| adiponectin | GCAGAGATGGCACTCCTGGA | CCCTTCAGCTCCTGTCATTCC |

**Supplemental Figures**


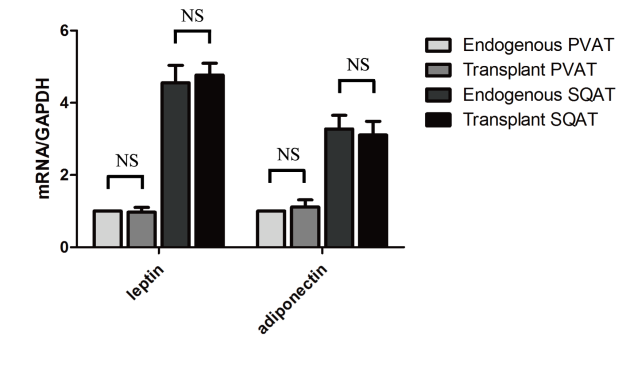


**Fig.S1** Comparison of expression of leptin and adiponectin in transplanted PV or SQ adipose tissue versus the corresponding endogenous adipose tissues from recipient mice. Tissues were pooled from 6 animals and run in triple to obtain these results due to the small volume of transplanted adipose tissues

**
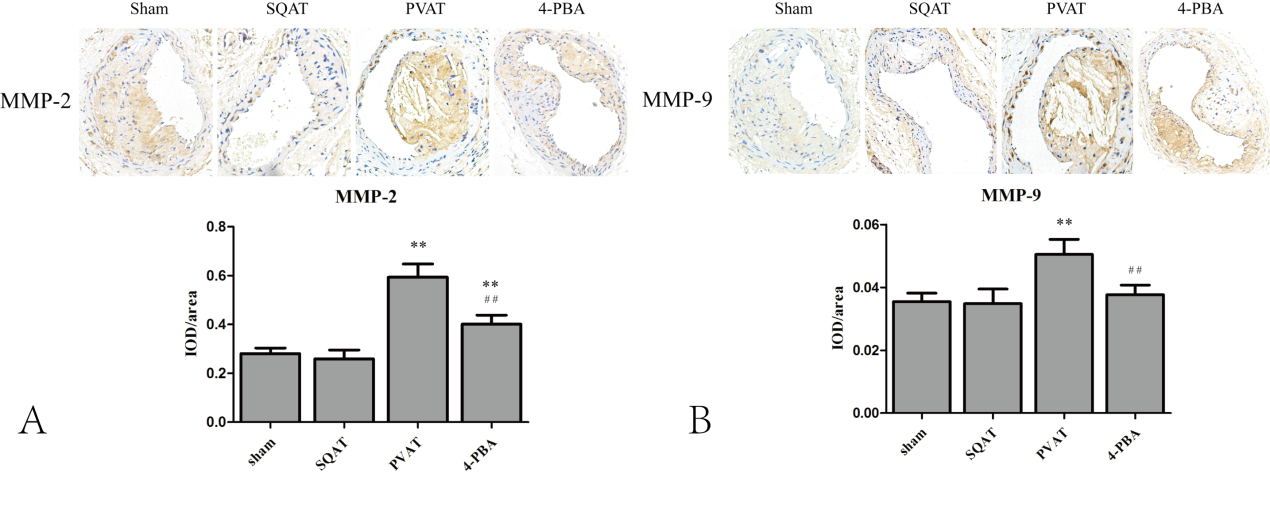
**

**Fig.S2** Effects of adipose tissue transplantation on the MMP2/9 expression in 4 groups of mice. A. Immunostaining for MMP2 expression. B. Immunostaining for MMP9 expression. n=6. ***p*<0.01 compared with sham group，^##^*p*<0.01 compared with PVAT group
